# Ménétrier’s disease, a premalignant condition, with coexisting advanced gastric cancer: A case report and review of the literature

**DOI:** 10.3892/ol.2014.2141

**Published:** 2014-05-13

**Authors:** ANNA PRYCZYNICZ, ROMAN BANDURSKI, KATARZYNA GUZIŃSKA-USTYMOWICZ, KATARZYNA NIEWIAROWSKA, ANDRZEJ KEMONA, BOGUSŁAW KĘDRA

**Affiliations:** 1 Department of General Pathomorphology, The Medical University of Białystok, Białystok 15-269, Poland; 2 The Second Department of General and Gastroenterological Surgery, The Medical University of Białystok, Białystok 15-269, Poland

**Keywords:** adenocarcinoma, gastric cancer, Ménétrier’s disease

## Abstract

Ménétrier’s disease (MD) is a rare type of hypertrophic gastropathy involving the body of the stomach, which is characterized by thickening of the mucous membrane in the form of giant rugal folds, hypochlorhydria and protein loss. The potential for malignant transformation of this lesion remains a controversial topic. Therefore, in the present study, a case of a 51-year-old male exhibiting MD with coexisting advanced gastric cancer is described; a review of the literature is also presented. The present case emphasized that MD requires particular attention and should be regarded as a premalignant condition due to the previously documented cases of its coexistence with gastric cancer, in addition to the lack of knowledge regarding its pathogenesis and effective therapeutic management.

## Introduction

Ménétrier’s disease (MD) is a rare type of hypertrophic gastropathy involving the body of the stomach, which was initially described in 1888 (1). It is characterized by thickening of the gastric mucosa in the form of giant rugal folds, hypochlorhydria and protein loss. The classic symptoms of MD include abdominal pain, nausea, vomiting and peripheral oedema (2). It occurs in two forms, depending on the patient’s age; in adult males aged ~55 years, it presents as a progressive disease with an asymptomatic onset. However, in early childhood, it develops abruptly and resolves spontaneously. While the development of MD is associated with the cytomegalovirus (CMV) infection in the case of infants, the cause of MD in adults remains unknown (3–5). Previous studies demonstrate the coexistence of MD with infections (*Helicobacter pylori*, CMV, herpes simplex, human immunodeficiency virus [HIV], *Mycoplasma pneumoniae*) (4,6–9) as well as non-specific inflammatory diseases (including ulcerative colitis) (10). However, the administration of targeted therapy in these disorders has not provided any benefits concerning the treatment of MD. Furthermore, the coexistence of MD with different types of cancer has been documented in >50 cases. It remains unknown as to whether cancer develops from MD, whether the disease is a premalignant condition and what the possible mechanism of carcinogenesis is. In the current study, a patient with MD and advanced gastric cancer is described and a review of the literature is presented, which indicates that MD should be recognized as a premalignant condition. The studywas approved by the ethics committee of the Medical University of Białystok, (Białystok, Poland) and the patient provided written informed consent.

## Case report

### History

**Figure 1:**
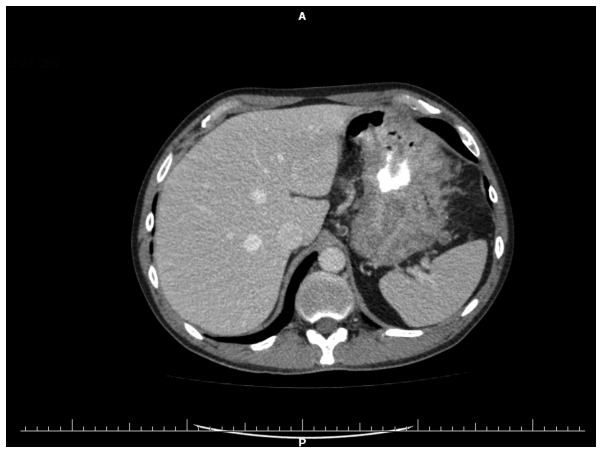
Multi-slice computed tomography examination of the stomach demonstrating the corpus and the prepyloric part with the heterogeneous wall thickened to 34.5 mm.

**Figure 2:**
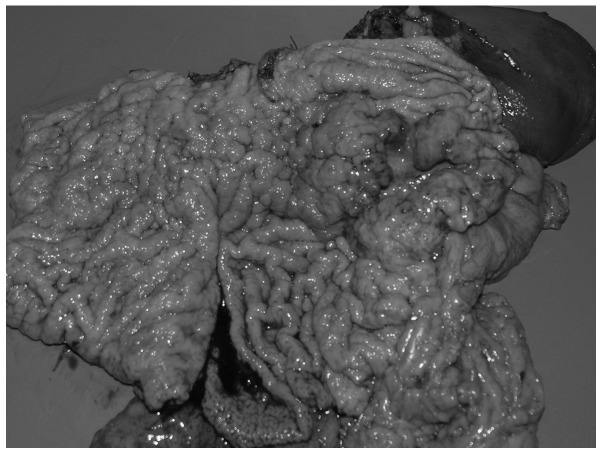
Gross postoperative specimen. The neoplastic infiltration diameter was 5 cm and a 35-mm diameter crater ulceration was located at the greater curvature of the stomach in the upper third. A thickened gastric wall with significantly oversized, rugal folds of the mucous membrane was observed.

A 51-year-old male was admitted to the Second Department of Surgery and Gastroenterology (Białystok, Poland) for planned surgery and was diagnosed with stomach cancer by performance of a gastroscopy. The description of the preoperative endoscopy, obtained by the patient from another center, demonstrated a nodular change with an irregular friable ulceration, localized on the border of the body and the fundus on the posterior wall of the stomach. The histopathological examination of the biopsy material identified that it was an adenocarcinoma. The patient’s medical history revealed only abdominal pain without weight loss, normal peristalsis and regular stools. The preoperative blood parameters showed no abnormalities. Only the α1-globulin level was marginally elevated to 0.24 g/dl, and the total protein concentration and albumin level were normal. The tumour markers, carcinoembryonic antigen and cancer antigen 19-9, were also normal. Multi-slice computed tomography of the stomach was performed preoperatively and demonstrated that the heterogenic wall within the corpus and the prepyloric part had thickened to 34.5 mm (Fig. 1). Intraoperatively, a large neoplastic infiltration was identified, ~5 cm in diameter, with a 35-mm crater ulceration at the greater curvature of the stomach, in the upper third, infiltrating the pancreatic tail and involving the splenic flexure. The gastric wall was thickened and showed massive, oversized rugal folds of the mucous membrane. In addition, the regional lymph nodes of the stomach were markedly enlarged. The splenic flexure was separated from the tumour. A total gastrectomy was conducted to remove the spleen and pancreatic tail and the lymph nodes were removed by D2 lymphadenectomy. The digestive tract was reconstructed using the double tract reconstruction technique that enables the passage of chyme through the duodenum. The postoperative period was uneventful (Fig. 2).

### Histopathology

Macroscopically, the postoperative formalin-fixed sample showed a tumour situated on the anterior wall of the stomach, partly on the lesser curvature, which was 10 cm at the greatest diameter. An additional lesion was identified on the posterior wall of the stomach, located 3 cm away from the other tumour. The tumours were characterized by exophytic growth, were poorly demarcated and the surrounding mucous membrane was infiltrated.

**Figure 3:**
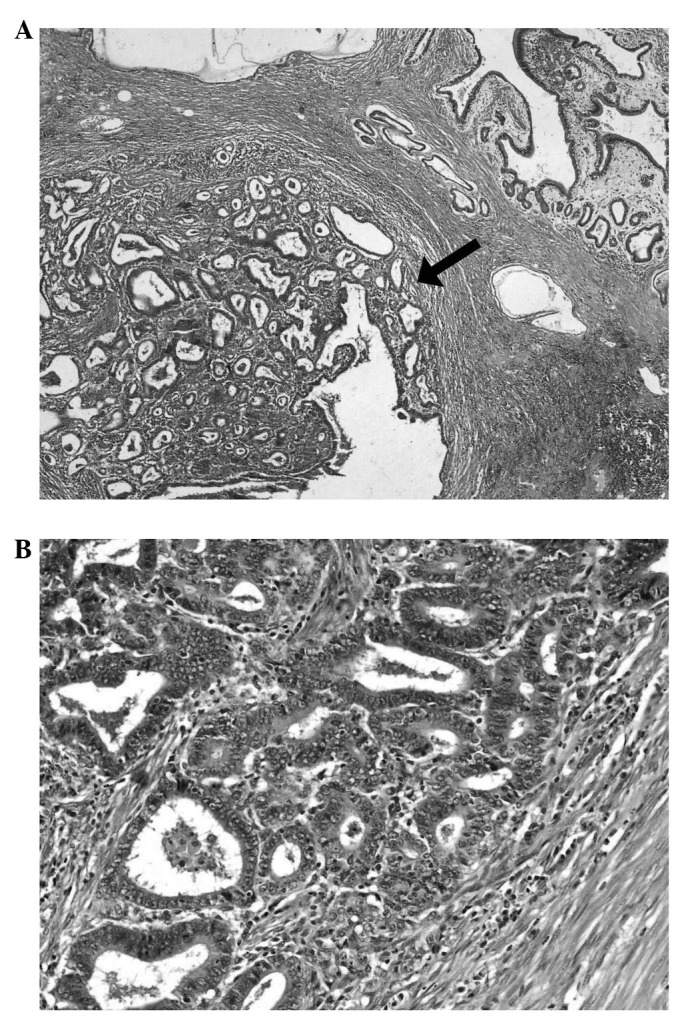
(A) Cancer (arrow) adjacent to the site of Ménétier’s disease (Hematoxylin and eosin [H&E] staining; magnification ×20). (B) Higher magnification of moderately differentiated tubular adenocarcinoma (H&E staining; magnification ×40).

The microscopic examination of the first lesion demonstrated tubular and papillary adenocarcinoma and was classified histologically using the 7th edition of the Union for International Cancer Control classification (11) as extending to the serosal mucosa (pT3) with a moderately differentiated malignancy (G2) (12). The tumour was classified as an intestinal type, according to Lauren’s classification, and as type I according to the Goseki criteria (numerous glandular structures and a small quantity of mucus in the cancer cells). The immunohistochemical analysis for human epidermal growth factor receptor 2 was negative, and metastases to 21/78 lymph nodes and tumour infiltration of the pancreatic tail were observed (Fig. 3).

**Figure 4:**
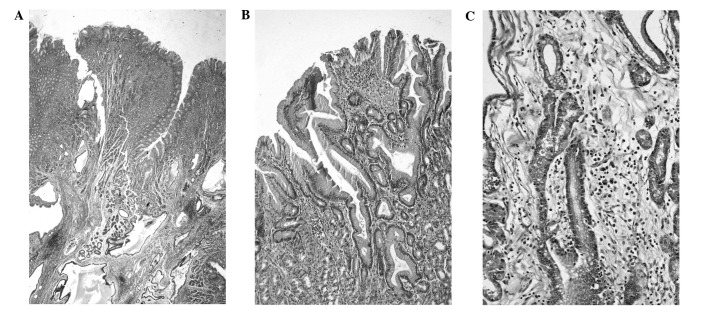
Ménétrier’s disease. (A) Hyperplastic change of the epithelium with cystic dilatation of the gastric glands (Hematoxylin and eosin [H&E] staining; magnification, ×10). (B) Tortuosity of the gastric glands and foveolar hyperplasia (H&E staining; magnification ×20). (C) Infiltration of eosinophils (H&E staining; magnification, ×40).

The microscopic image of the smaller lesion substantiated the diagnosis of MD. The gastric mucosa was thickened and appeared polyp-like. The mucosal architecture was normal with glands remaining parallel at the surface. However, minimal architectural disorder was identified in the deep third of the mucosa. Foveolar hyperplasia was poorly marked and the tortuosity of the glands was localized in the upper and middle third of the mucosa. Cystic dilatation of the glands was distinct in the lower regions of the mucosa and submucosa. There was an increase in the number of parietal cells observed, and inflammatory infiltration predominantly consisted of numerous eosinophils and plasma cells. In addition, strongly pronounced smooth muscle hyperplasia and slight oedema were observed. The *H. pylori* infection was not detected (Fig. 4).

## Discussion

Since MD is rare and difficult to discriminate from other hypertrophic gastropathies, Rich *et al* (2) proposed an algorithm for its recognition. According to the algorithm, the diagnosis of MD should be based on a comprehensive collection of data concerning clinical, endoscopic, laboratory and histopathological findings. The most common symptoms of MD include abdominal pain, nausea, vomiting and oedema, in addition to serum albumin loss. Endoscopically, histopathological examination demonstrates a thickened gastric mucosa. Additionally, the gastric pH must be evaluated; in MD the pH is often alkaline. The characteristic microscopic features are as follows: Parallel mucosal gland ducts, tortuosity and dilatation, foveolar hyperplasia, a reduced number of parietal cells, infiltration of eosinophils and/or plasma cells, smooth muscle hyperplasia and oedema. The laboratory assessments, including complete blood count, serum protein, serum gastrin as well as serologic tests for *H. pylori* and CMV should also be performed (2). In the present case, the patient belonged to a group of MD patients with less pronounced clinical symptoms, who did not exhibit protein loss. The elevated number of parietal cells observed in the histopathological examination was the predominant difference. However, other characteristic features facilitated the diagnosis of MD.

As yet it has been impossible to determine the number of diagnosed cases of MD due to a lack of accurate incidence data. However, >50 cases of MD presenting with coexisting gastric cancer have been documented since 1983. Until 1990, ~30 cases had been described and Hsu et al (13) documented that a higher percentage of up to 18 cases of MD coexisted with early gastric cancer. Cases of MD coexisting with cancer documented after 1991 were collected as part of the present study; out of 16 MD cases, three were with early stage cancer, seven were with advanced stage cancer and six contained no evidence of the cancer stage (Table I) (14–24). Such statistics may be due to the patients’ origin. The majority of cases described by Hsu *et al* (13) originate from Japan, where screening for cancer is widely developed and early changes are more frequently diagnosed. Histologically, a predominance of poorly differentiated adenocarcinomas were identified during the literature search in the present study. In addition, in 2007, Choi *et al* (20) demonstrated a case of MD coexisting with squamous cell carcinoma. In the studies identified during the literature search, the patients were aged >40 years and nine out of the 12 cases described were male.

The risk of malignancy in MD was described in 1991 as 6–10% of the incidence rate (13). A premalignant condition is defined as a disease that may develop into a malignancy. This percentage is considered to be adequate to regard MD as a premalignant condition. Currently, it is not possible to determine and confirm this rate due to the low incidence of the disease and a lack of registration of all cases of MD.

In MD it is also unclear whether a tumour is formed on hyperplastic growth or develops as a *de novo* lesion. Usually, and in the cases that were presented in the literature, the two changes are diagnosed simultaneously during histological imaging. However, it was 1983 when Wood *et al* (25) described a man with MD who developed early gastric cancer 3.5 years following diagnosis. Furthermore, Ramia *et al* (19) reported a patient with advanced gastric cancer and MD, who was diagnosed with MD 13 years prior to the cancer diagnosis. In the abovementioned cases, the cancer developed subsequent to the diagnosis of MD. In the patient in the present study, the cancer was identified in the biopsy material, whereas MD was confirmed later in the postoperative specimen. Macroscopically, there were two distinct gastric tumours ~3 cm apart, which were connected to each other by infiltration that indicated independent growth. A morphological similarity of tumour growth to the cystic gland ducts in MD was observed. Histologically, the cancerous glands appeared to be enormous cystic formations with ingrowing papillary structures of cancer cells (Fig. 4). This provides further evidence for the hypothesis that cancer may arise from MD.

The poorly understood pathogenesis of MD that results in a lack of appropriate treatment strategies is another point in favour of recognising MD as a premalignant lesion. Currently, symptomatic treatment, anticholinergic agents, acid suppression, octreotides and prednisone are predominantly administered for its treatment (5). Furthermore, theories of bacterial (*H. pylori*, *Mycoplasma pneumoniae*) and viral infections (CMV, herpes simplex, HIV) have been proposed, which are associated with chronic inflammation of the gastric mucosa and result in further abnormal recovery in the form of hyperplasia (4–9). However, due to a lack of clinical trials, the benefits of targeted treatment for these infections in adults are not well known. A theory of increased epidermal growth factor receptor signalling associated with transforming growth factor-α overproduction has also been presented and treatment with monoclonal antibodies has been proposed (2). However, the optimum therapeutic procedure for MD is a gastrectomy, specifically in those patients with uncontrolled protein loss, bleeding and a high risk for coexisting cancer. A partial or total gastrectomy is performed depending on anastomosis, clinical observations and the cancer stage (17).

In conclusion, MD should be treated with particular attention and regarded as a premalignant condition due to the previously documented cases of its coexistence with gastric cancer, as well as the lack of knowledge regarding its pathogenesis and effective therapeutic management. This condition should not be overlooked, and the clinician and the patient should be aware that it is necessary to monitor the lesion via regular endoscopic biopsy examinations.

## Figures and Tables

**Table I:** Cases of Ménétrier’s disease presenting with gastric carcinoma since 1990.

			Patient characteristics				
			
Case	Author (ref.)	Year	Age (years)	Gender	Protein loss	Histology	Depth of invasion and metastases
1	Mosnier *et al* (14)	1991	63	M	+	Adenocarcinoma, G2	T - No data, N0
2	Johnson *et al* (15)	1995	73	M	No data	Adenocarcinoma	Early
3	Charton-Bain *et al* (16)	2000	62	F	No data	Adenocarcinoma, G1	Advanced - T2, N0
4	Kim *et al* (17)	2004	47	M	+	Adenocarcinoma, G1	Early - T1, N0
5–8	Sâadia *et al* (18)	2005	58–81^a^	No data	No data	Adenocarcinoma	No data
9	Ramia *et al* (19)	2007	66	M	+	Adenocarcinoma	Advanced, M1 (liver)
10	Choi *et al* (20)	2007	40	M	−	Squamous cell carcinoma, G3	Advanced - T4, N0
11	Salinas Martín *et al* (21)	2008	40	F	No data	Adenocarcinoma, G3	Advanced, N1
12	Salinas Martín *et al* (21)	2008	61	M	No data	Adenocarcinoma, G3	Advanced, N1
13	Mellado-Castillero *et al* (22)	2008	40	F	No data	Adenocarcinoma, G3	Advanced, N1
14	Pereyra *et al* (23)	2011	72	M	No data	Adenocarcinoma, G3	Early
15	Famularo *et al* (24)	2011	73	M	+	Poorly differentiated carcinoma	No data
16	Present case	2014	51	M	−	Adenocarcinoma, G2	Advanced - T3, N1

a Four different cases.

M, male; F, female; +, protein loss observed; -, protein loss not observed; G1, well-differentiation; G2, moderately differentiated; G3, poorly differentiated; T, direct extent of the primary tumor; N, lymph node metastasis; M, distant metastasis.
